# Suppressions of Migration and Invasion by Cantharidin in TSGH-8301 Human Bladder Carcinoma Cells through the Inhibitions of Matrix Metalloproteinase-2/-9 Signaling

**DOI:** 10.1155/2013/190281

**Published:** 2013-01-27

**Authors:** Yi-Ping Huang, Chien-Hang Ni, Chi-Cheng Lu, Jo-Hua Chiang, Jai-Sing Yang, Yang-Ching Ko, Jing-Pin Lin, Jehn-Hwa Kuo, Shu-Jen Chang, Jing-Gung Chung

**Affiliations:** ^1^Department of Physiology, China Medical University, Taichung 404, Taiwan; ^2^Department of Chinese Medicine, E-DA Hospital/I-Shou University, Kaohsiung 824, Taiwan; ^3^Department of Life Sciences, National Chung Hsing University, Taichung 402, Taiwan; ^4^Department of Pharmacology, China Medical University, Taichung 404, Taiwan; ^5^Division of Pulmonary and Critical Care Medicine, Department of Internal Medicine, St. Martin De Porres Hospital, Chiayi 600, Taiwan; ^6^School of Chinese Medicine, China Medical University, Taichung 404, Taiwan; ^7^Special Class of Healthcare, Industry Management, Central Taiwan University of Science and Technology, Taichung 406, Taiwan; ^8^Department of Urology, Jen-Ai Hospital, Taichung 412, Taiwan; ^9^School of Pharmacy, China Medical University, Taichung 404, Taiwan; ^10^Department of Biological Science and Technology, China Medical University, Taichung 404, Taiwan; ^11^Department of Microbiology, China Medical University, Taichung 404, Taiwan; ^12^Department of Biotechnology, Asia University, Taichung 413, Taiwan

## Abstract

Cancer metastasis becomes an initial cause of cancer death in human population. In many cancers, it has been shown that the high levels of matrix metalloproteinase (MMP)-2 and/or MMP-9 are associated with the invasive phenotypes of cancer cells. In this study, we investigated the effects of cantharidin, a derivative of *blister beetles* which is one of the traditional Chinese medicines, on the adhesion, migration, and invasion of human bladder cancer TSGH-8301 cells. Cantharidin effectively suppressed TSGH-8301 cell adhesion, migration, and invasion in a concentration-dependent manner. Results from Western blotting, RT-PCR, and gelatin zymography assays indicated that cantharidin blocked the protein levels, gene expression (mRNA), and activities of MMP-2 and -9 in TSGH-8301 cells. Cantharidin also significantly suppressed the protein expressions of p-p38 and p-JNK1/2 in TSGH-8301 cells. Taken together, cantharidin was suggested to present antimetastatic potential *via* suppressing the levels of MMP-2 and MMP-9 expression that might be mediated by targeting the p38 and JNK1/2 MAPKs pathway in TSGH-8301 human bladder cancer cells.

## 1. Introduction

In genitourinary tumor, bladder cancer is a significant cause of morbidity and mortality [1]. In the United States, bladder cancer is the fourth most common malignancy, and new cases about 70,530 (52,760 men and 17,770 women) and deaths for the year 2010 were 14,680 (10,410 men and 4270 women) [2]. In Taiwan, about 2.3 individuals per 100,000 die annually from bladder cancer on the basis of the 2011 report from the Department of Health, Taiwan. In bladder cancer of patients, 75% present with superficial disease and 25% with invasive disease [3].

During the metastasis development, there are about 50% of patients with muscle invasive bladder cancer within 2 years of cystectomy [4, 5]. Muscle-invasive bladder cancer is an aggressive epithelial tumor; almost 50% of these patients develop metastases and ultimately succumb to their disease with poor long-term survival [6, 7]. Invasion and metastasis are predominant properties in cancer cells that led to hard-to-cure patients [8, 9]. It is well documented that the activities of matrix metalloproteinases (MMPs) play an important role in the cancer cell's metastasis process, including cell adhesion, migration, and invasion [10–12]. Therefore, blockage of the activities of MMPs may be a strategy to inhibit the cancer cell metastasis.

Cantharidin, a derivative of *Blister Beetles*, is protein phosphatase 1 (PP1) and protein phosphatase 2A (PP2A) inhibitors [13, 14] and has been used in traditional Chinese medicine [15]. Cantharidin induced cell cycle arrest [16, 17] and triggered apoptosis in various types of tumor cells, including hepatoma [18], myeloma [19], oral buccal carcinoma [20], leukemia cells [21, 22], and intestinal epithelial cells [23]. Recently, cantharidin was found in our laboratory to provoke apoptosis in human bladder carcinoma TSGH-8301 and colorectal cancer colo 205 cells [24, 25] but there is no report to show that cantharidin inhibited the migration and invasion of TSGH-8301 cells. Therefore, the current study investigated the effects of cantharidin on migration and invasion and explored its signaling molecules in *in vitro* study. Our results demonstrated that cantharidin potently inhibited the migration and invasion of TSGH-8301 human bladder carcinoma cells through inhibiting the p38 and JNK1/2-modulated MMP-2/-9 signaling *in vitro*.

## 2. Materials and Methods

### 2.1. Chemicals and Reagents

Cantharidin, dimethyl sulfoxide (DMSO), propidium iodide (PI) and anti-*β*-Actin were purchased from Sigma-Aldrich Corp. (St. Louis, MO, USA). Cantharidin was dissolved in DMSO at a stock concentration of 50 mM and followed to dilute in further experiments. RPMI-1640 medium, L-glutamine, fetal bovine serum (FBS), penicillin-streptomycin, and trypsin-EDTA were obtained from Gibco/Life Technologies (Grand Island, NY, USA). Anti-MMP-9 (Cat. AB19016) and Millicell Hanging Cell Culture Inserts (Cat. PIEP12R48) were brought from Merck Millipore Corp. (Billerica, MA, USA). The antibodies to p-p38, p-JNK1/2, p-ERK1/2, and MMP-2 and horseradish-peroxidase- (HRP-) conjugated secondary antibodies were purchased from Santa Cruz Biotechnology, Inc. (Santa Cruz, CA, USA).

### 2.2. Cell Culture

The human bladder carcinoma TSGH-8301 cell line was purchased from the Food Industry Research and Development Institute (Hsinchu, Taiwan). TSGH-8301 cells were maintained in RPMI-1640 medium supplemented with 10% FBS, 100 Units/mL penicillin, and 100 *μ*g/mL streptomycin in 75 cm^2^ tissue culture flasks and grown at 37°C under a humidified atmosphere with 5% CO_2_ as previously described [24, 26].

### 2.3. Assessment for Cell Viability

TSGH-8301 cells were seeded at a density of 2 × 10^5^ cells/well in 12-well plates and were incubated with 0, 0.25, 0.5, 1, 2, and 2.5 *μ*M of cantharidin for 24 h. DMSO at the concentration of 0.5% served as a vehicle control. Cells were harvested and were stained with PI (5 *μ*g/mL) and then were analyzed by flow cytometry (BD Biosciences, FACSCalibur, San Jose, CA, USA) for viability determinations as previously described [24, 27].

### 2.4. Adhesion Assay

TSGH-8301 cells at the density of 5 × 10^4^ cells/well were preincubated with cantharidin (0, 1, and 2.5 *μ*M) and 0.5% DMSO (vehicle control) for 24 or 48 h at 37°C in 96-well plates precoated with type I collagen (10 *μ*g/mL) (EMD Millipore) for 60 min at 37°C. After a 3 h incubation, the unattached cells were removed, and attached cells were fixed in 1% glutaraldehyde in PBS for 20 min. Then cells were stained with 0.02% crystal violet solution for 5 min at room temperature. For quantification of the attached cells, 70% ethanol was used to dissolve the crystal violet, and O.D. was measured at 570 nm by using microplate reader and reference 405 nm. The percentage of adhesion was calculated based on the adhesion cells compared to control [28, 29].

### 2.5. Wound Healing Assay

TSGH-8301 cells at the density of 5 × 10^5^ cells/well were maintained in 6-well plates and incubated at 37°C for 24 h. After cells were grown in confluent then cells were scratched with a 200-*μ*L pipette tip, cells in the plate were washed with PBS, and then added the new complete medium then were treated with or without 1 and 2.5 *μ*M of cantharidin for 24 h and 0.5% DMSO served as a vehicle control. At the end of incubation, the cells were examined and were photographed under a fluorescence microscope. The number of cells that migrated into the scratched area was calculated as described elsewhere [28, 30].

### 2.6. *In Vitro* Migration and Invasion Assays

TSGH-8301 cell migration or invasion was conducted using 24-well Transwell inserts (8 *μ*m pore filters, Merck Millipore) individually coated with 30 *μ*g type I collagen (Merck Millipore) (for migration) or Matrigel (BD Biosciences, Bedford, MA, USA) (for invasion) [28]. In brief, TSGH-8301 cells (2 × 10^4^ cells/well) were cultured for 24 h in serum-free RPMI-1640 medium, and then cells were placed in the upper chamber of the Transwell insert and treated with 0.5% DMSO (as a control) or cantharidin (1 or 2.5 *μ*M) for 24 h. In the lower chamber, the medium containing 10% FBS was placed. At the end of incubation, the nonmigrated cells were removed using a cotton swab; the invaded cells maintained in the upper chamber were fixed with 4% formaldehyde and stained with 2% crystal violet. In the lower surface of the filter, cells penetrated were counted and photographed under a phase-contract microscope at a 200x magnification. Three independent experiments were performed as described elsewhere [31, 32].

### 2.7. Western Blotting Analysis

For investigating the protein levels associated with migration and invasion, whether are affected or not by cantharidin, we determined related signaling molecules by Western blotting as described elsewhere [27, 33, 34]. Briefly, TSGH-8301 cells (1 × 10^6^ cells/well) were placed in 6-well plates for 24 h and then were incubated with cantharidin (0, 1, or 2.5 *μ*M) for 24 h. At the end of incubation, cells were harvested from each treatment and were individually lysed in lysis buffer (PRO-PREP protein extraction solution, iNtRON Biotechnology, Seongnam-si, Gyeonggi-do, Korea). The total protein amount was individually determined using a Bio-Rad protein assay kit (Bio-Rad Laboratories, Hercules, CA, USA). The protein abundance of p-p38, p-JNK1/2, p-ERK1/2, MMP-2, and MMP-9 was examined by sodium dodecyl sulfate-polyacrylamide gel electrophoresis (SDS-PAGE) and Western blotting as previously described [33, 34]. The relative abundance of each band which represents associated protein expression was quantified using the NIH ImageJ [35].

### 2.8. Gelatin Zymography Assay

TSGH-8301 cells at the density of 1 × 10^6^ cells/well were plated in 12-well plates and then were incubated in serum-free RPMI-1640 medium in the presence of 0, 1, or 2.5 *μ*M of cantharidin for 24 and 48 h. In the end of incubation, the conditioned medium was harvested, placed on 10% SDS-PAGE containing 0.2% gelatin (Sigma-Aldrich Corp.), and then separated by electrophoresis. The gels were soaked in 2.5% Triton X-100 in dH_2_O twice for a total of 60 min at 25°C to remove SDS. Gels were incubated at 37°C with substrate buffer (50 mM Tris HCl, 5 mM CaCl_2_, 0.02% NaN_3_, and 1% triton X-100, pH 8.0) for 18 h. The gel was stained using 0.2% Coomassie blue for 1 h, was destained in water containing 10% acetic acid and 50% methanol, and bands corresponding to the activity of MMP-2 and -9 were quantified with the NIH ImageJ software as previously described [28, 36].

### 2.9. Real-Time PCR of MMP-2 and -9 mRNA Expressions

TSGH-8301 cells at the density 1 × 10^6^ cells/well were placed in 6-well plates for 24 h and then were incubated with cantharidin (0, 1, or 2.5 *μ*M) for 24 h. Cells from each treatment were harvested and total RNA was extracted as previously described [33]. RNA samples were reverse-transcribed at 42°C with High Capacity cDNA Reverse Transcription Kit for 30 min according to the protocol of the supplier (Applied Biosystems, Foster City, CA, USA). The primers were set as MMP-2F: CCCCAGACAGGTGATCTTGAC; MMP-2R: GCTTGCGAGGGAAGAAGTTG; MMP-7F: GGATGGTAGCAGTCTAGGGATTAACT; MMP-9F: CGCTGGGCTTAGATCATTCC; MMP-9R: AGGTTGGATACATCACTGCATTAGG; GAPDH-F: ACACCCACTCCTCCACCTTT; GAPDH-R: TAGCCAAATTCGTTGTCATACC. Each assay was performed in triplicate by using the Applied Biosystems 7300 Real-Time PCR system, and the expression fold changes were performed by using the comparative C_*T*_ (threshold cycle) method [31, 34].

### 2.10. Statistical Analysis

Statistical differences were determined using one-way analysis of variance (ANOVA) followed by Dunnett's posttest and considered significant at the *P* < 0.05 between experimental and control samples. All data are presented as means ± standard deviation (SD) in triplicate of at least three independent experiments.

## 3. Results

### 3.1. Cantharidin Has No Effect on Percentage of Viable TSGH-8301 Cells

**Figure 1:**
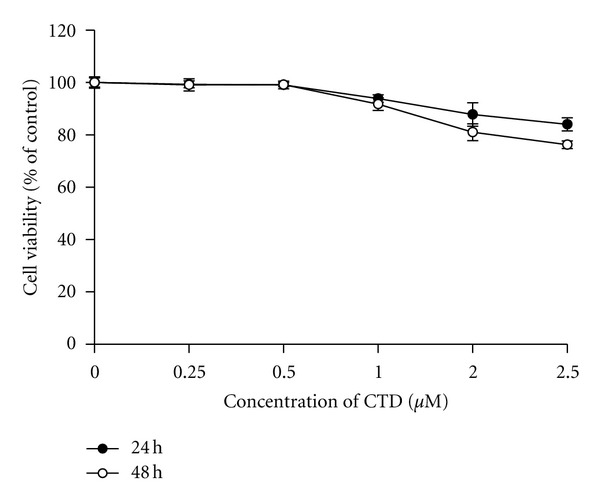
Cantharidin has no dramatic influence on the percentage of viable TSGH-8301 cells. Cells in 12-well plate were incubated with or without 0, 0.25, 0.5, 1, 2, and 2.5 *μ*M of cantharidin for 24 h and 48 h and then harvested for determination of the percentage of viable cells by flow cytometry as described in Section 2. Data represents mean ± S.D. in triplicate.

It is well documented that cantharidin decreased the percentage of viable cells in many types of human cancer cell lines [18–20, 22, 23]. TSGH-8301 cells were treated with various concentrations of cantharidin in serum-containing medium for 24 and 48 h, and cell viability was determined by flow cytometric assay. Results are shown in Figure 1 and indicated that cantharidin slightly decreased cell viability at the concentration of 1 *μ*M but cantharidin at 2.5 *μ*M decreased cell viability by approximately 17% and 30% at 24 and 48 h, respectively (Figure 1).

### 3.2. Cantharidin Decreases Cell Adhesion of TSGH-8301 Cells

**Figure 2:**
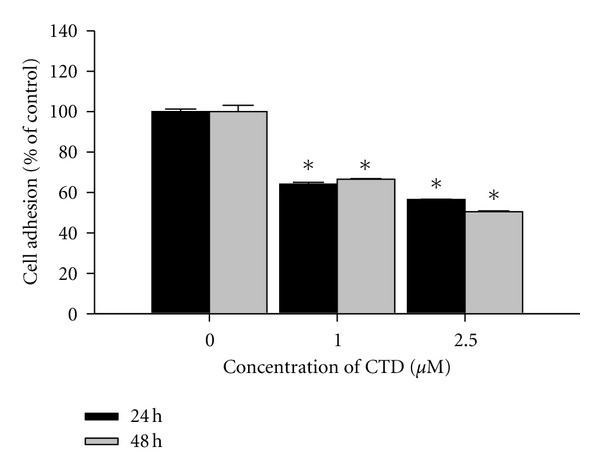
Cantharidin decreases cell adhesion of TSGH-8301 cells. Cells were incubated with or without 0, 1, and 2.5 *μ*M of cantharidin for 24 h and 48 h; then cells were measured the percentage of cell adhesion as described in Section 2. Data represents mean ± SD in triplicate and *P < 0.05, significant difference between cantharidin-treated groups and the control as analyzed by Dunnett's posttest.

To investigate the effects of cantharidin on the adhesion of TSGH-8301 cells, adhesive cells were quantified, and results are demonstrated in Figure 2. TSGH-8301 cells after incubation with cantharidin at the final concentrations (0, 1, and 2.5 *μ*M) for 24 and 48 h indicated that cantharidin significantly inhibited cell adhesion in a concentration- and time-dependent manner. Approximately 52% and 58% reduction were seen within 2.5 *μ*M treatment for 24 and 48 h, respectively.

### 3.3. Cantharidin Blocks TSGH-8301 Cell Migration by Wound Healing Examination

**Figure 3:**
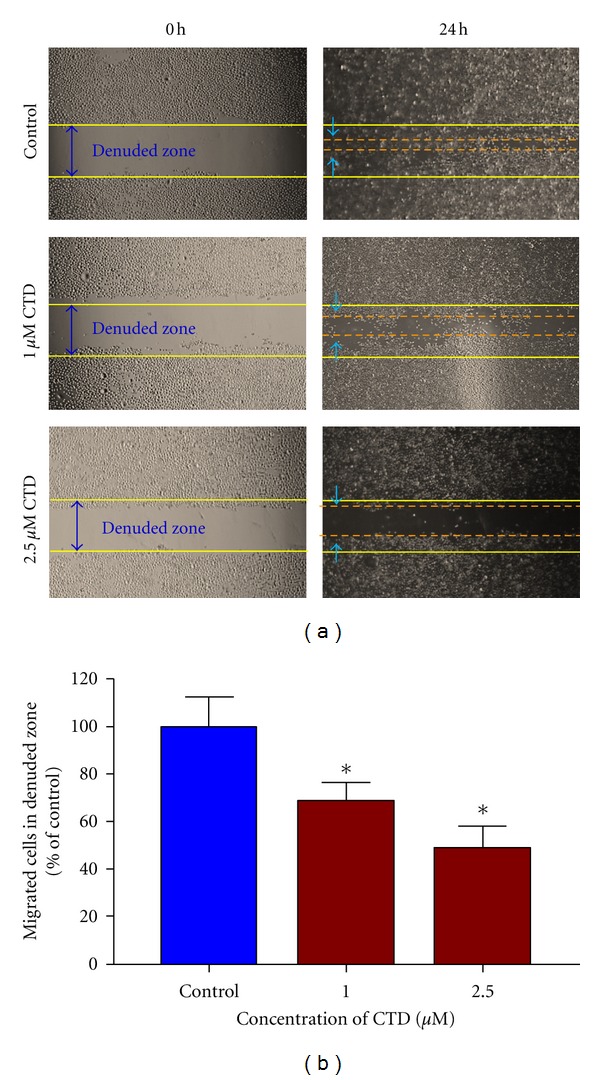
Wound healing assay for the effects of cantharidin on the migration of TSGH-8301 cells. Cells were placed for 24 h; then a wound was performed by scraping confluent cell layers with a pipette tip. Cantharidin was added to the cells at the final concentration were 0, 1 and 2.5 *μ*M and then incubated for 24. (a) Some of the representative photographs of invading treated and untreated cells are presented. Three separate experiments with similar results were carried out. (b) The migrated cells in the five random fields after exposure for 24 h were counted to quantify, and data was expressed as mean ± S.D. on the basis of untreated cells (control) represented as 100%. *P < 0.05, significant difference between cantharidin-treated groups and the control as analyzed by Dunnett's posttest.

Since data in Figure 2 indicated that cantharidin inhibited the adhesion of TSGH-8301 cells, we used wound-healing assay to examine the inhibition of cell migration of TSGH-8301* in vitro*.  Figure 3 displays that the migration distance between the leading edge and the wound line was compared between cantharidin-treated and untreated cells (Figure 3(a)). The results demonstrated that cantharidin suppressed the migration of TSGH-8301 cells in a concentration-dependent manner (Figure 3(b)).

### 3.4. Cantharidin Inhibits the Migration and Invasion of TSGH-8301 Cells *In  Vitro*

**Figure 4:**
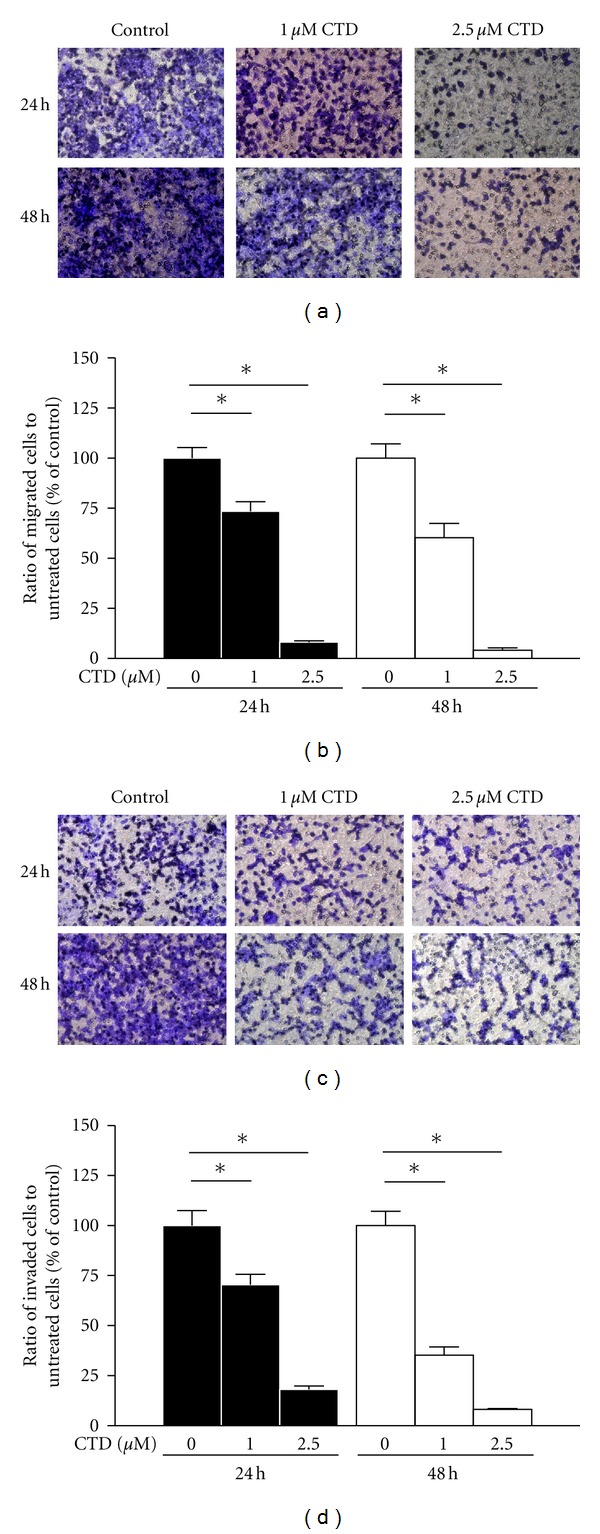
Cantharidin inhibits the migration and invasion of TSGH-8301 cells *in vitro*. Cells were placed in the well at the density of  5 × 10^4^ cells/well and then were treated with 0, 1, and 2.5 *μ*M cantharidin then that penetrated through with or without the Matrigel to the lower surface of the filter were examined. Cells were stained with crystal violet and then were examined and photographed under a light microscope at 200x (a and c). The quantification of cells from each treatment in the lower chambers was counted at 200x (b) and (d). Columns repeat the mean from three independent experiments. *P < 0.05, significant difference between cantharidin-treated groups and the control as analyzed by Dunnett's posttest.

For further investigating if cantharidin inhibits the migration and invasion of TSGH-8301 cells, Boyden chamber assay was performed and results are shown in Figures 4(a), 4(b), 4(c), and 4(d). These results were obtained due to the effects of cantharidin on cell migration (Figures 4(a) and 4(b)) and invasion (Figures 4(c) and 4(d)) in TSGH-8301 cells that were treated with 0, 1, and 2.5 *μ*M of cantharidin for 24 and 48 h (cell migration and invasion). Results indicated that cantharidin reduced the migration and invasion of TSGH-8301 cells substantially in a concentration-dependent manner.

### 3.5. Cantharidin Affects the Levels of Associated Protein and Gene Levels for Migration and Invasion of TSGH-8301 Cells

**Figure 5:**
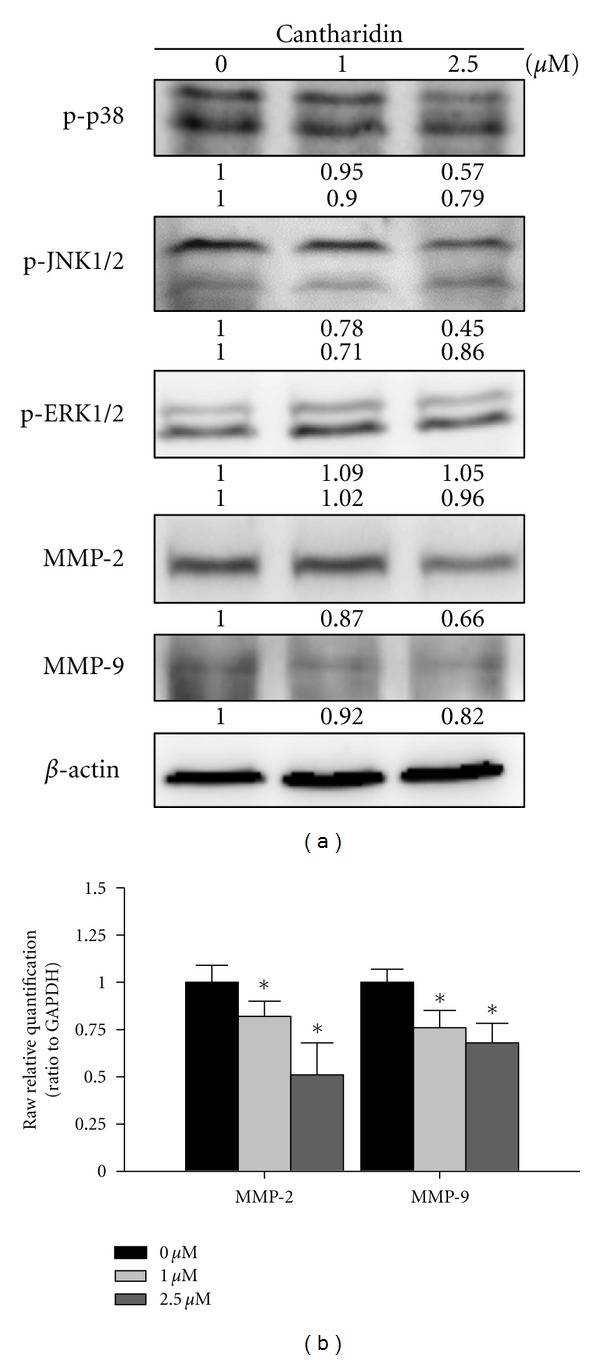
Cantharidin affects the levels of associated proteins and gene levels in migration and invasion of TSGH-8301 cells. Cells were treated with cantharidin (0, 1, and 2.5 *μ*M) for 24 h, and then cells were collected. The total protein extract was quantified and determined as described in Section 2. The levels of p-p38, p-JNK1/2, p-ERK1/2, MMP-2, and MMP-9 protein expressions (a) were estimated by Western blotting as described in Section 2. (b) The total RNA was extracted from cantharidin-treated cells, and the RNA samples were reverse-transcribed to cDNA for real-time PCR as described in Section 2. The ratios between MMP-2, MMP-9, and GAPDH mRNA are used and data represents mean ± SD in duplicate of at least three independent experiments. *P < 0.05 was considered significantly as analyzed by Dunnett's posttest.

We further examined the effects of cantharidin on the inhibition of migration and invasion of TSGH-8301 cells, which are involved in the effects of associated protein levels of migration and invasion; those changes of associated protein were measured by SDS-PAGE and Western blotting. TSGH-8301 cells were treated with cantharidin (0, 1, and 2.5 *μ*M) for 24 h and then subjected to Western blotting, and results are shown in Figure 5(a). Results from Western blotting showed that cantharidin could reduce the phosphorylation of p38 and JNK1/2 as well as MMP-2 and -9 in TSGH-8301 cells. However, the protein level of p-ERK1/2 was no significant alteration in comparison to untreated control.  Figure 5(b) indicated that cantharidin suppressed the gene expression of MMP-2 and -9 in TSGH-8301 cells.

### 3.6. Cantharidin Suppresses the Activities of Matrix Metalloproteinases (MMPs) in TSGH-8301 Cells

**Figure 6:**
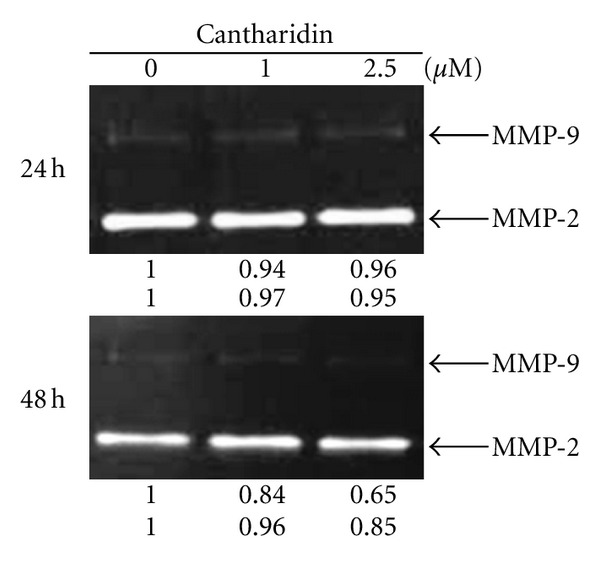
Cantharidin suppresses the activities of matrix metalloproteinases (MMPs) in TSGH-8301 cells. Gelatin zymography was used to evaluate the activities of MMP-2 and MMP-9 as described in Section 2. The different activity of MMP-2 and -9 was determined by densitometry analysis, and results are expressed as % of control. Similar results were obtained from three independent experiments.

Gelatin zymography was used for analysis of MMP-2 and -9 activities. As shown in Figure 6, cantharidin treatment may lead to reduced activity of MMP-2 and -9 in a dose-dependent manner. This also confirmed that cantharidin inhibited the gene expression (mRNA) of MMP-2 and -9 in TSGH-8301 cells (Figure 5(b)).

## 4. Discussion

Numerous reports have demonstrated that cantharidin processes antitumor properties [16–23] but there is no report to show the inhibition of migration and invasion of human bladder cancer cells. In this study, we investigated the inhibitory effects of cantharidin on the adhesion, invasion, and migration of TSGH-8301 cells. Results indicated that cantharidin inhibited the cell adhesion, invasion and migration on TSGH-8301 cells (Figures 2 and 3). Cantharidin decreased the protein expressions, gene expression (mRNA) and activities of MMP-2 and -9 in TSGH-8301 (Figures 5(a) and 5(b) and 6). In general, metastasis of cancer cells involves multiple processes, proteins function, and various physiological changes. Furthermore, the degradation or breakdown of the ECM through protease is a critical step in tumor invasion or migration [29, 30]. The involved proteases in migration and invasion, in particular, MMP-2 and MMP-9 were reported to play an important role in cancer invasion and metastasis [37, 38].

Mitogen-activated protein kinases (MAPKs) include p38, c-Jun-*N*-terminal kinase (JNK), and extracellular signal-regulated kinase (ERK) [35], and MAPKs activation is followed by phosphorylation of a variety of cytosolic substrates associated with cell proliferation, cell differentiation, cell invasion, cell migration, and cell death [39, 40]. It was reported that MAPK pathways were involved in the regulation of MMPs and uPA expression in tumor-cell invasion [41, 42]. Herein, we verified that cantharidin has an inhibitory effect on migration and invasion through the suppression of MMP-2 and -9 in TSGH-8301 cells. We further found that cantharidin inhibited the p-JNK1/2 and p-p38. Thus, our results suggested that cantharidin downregulated MMP-2 and MMP-9 protein expression and suppressed metastatic effect through JNK1/2 and p38 MAPKs signals but not ERK1/2 molecule on TSGH-8301 cells.

**Figure 7:**
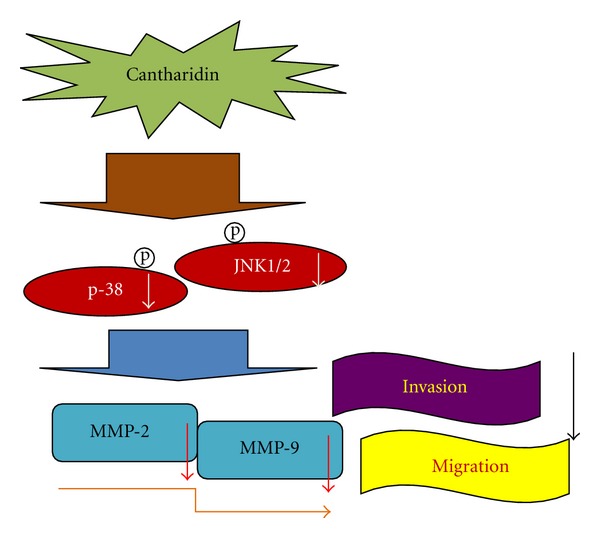
The possible working model and signaling transduction molecules for cantharidin-inhibited cell invasion and migration of TSGH-8301 human bladder cancer cells.

Taken together, the present study showed novel findings addressing that cantharidin exerts an inhibitory effect on several essential steps of cancer cell metastasis, including cell adhesion, invasion, and migration *via* regulating the activities of metastasis-associated proteases such as MMP-2 and -9. Based on those observations, we suggest that cantharidin could be a powerful candidate for development of preventive agents against bladder cancer metastasis in the future. Overall, we showed that cantharidin effectively inhibits the expressions of p-p38 and p-JNK1/2, causing downregulation of MMP-2 and -9 in TSGH-8301 cells that can be seen in Figure 7. Thus, cantharidin could be tested further *in vivo *to justify its effectiveness in the prevention of bladder tumor cell invasion or migration during cancer treatment.
